# Redefining the Treatment Landscape of Advanced Endometrial Cancer in the Era of Immunotherapy and Precision Oncology

**DOI:** 10.3390/cancers18111837

**Published:** 2026-06-04

**Authors:** Martina Cassaniti, Ilaria Morelli, Anna Chiara Boschi, Simona Scodes, Giuseppe Comerci, Claudia Casanova, Stefano Tamberi

**Affiliations:** 1 Oncology Unit, Santa Maria Delle Croci Hospital, AUSL Della Romagna, 48121 Ravenna, Italy; martina.cassaniti@auslromagna.it (M.C.); simona.scodes@auslromagna.it (S.S.); claudia.casanova@auslromagna.it (C.C.); stefano.tamberi@auslromagna.it (S.T.); 2 Oncology Unit, University Hospital of Ferrara, 44124 Ferrara, Italy; 3 Department of Medical Sciences (DIMEC), University of Bologna, 40126 Bologna, Italy; 4 Obstetrics and Gynecology, Hospital S. Maria delle Croci, Ravenna, 48100 Ravenna, Italy; annachiara.boschi@auslromagna.it (A.C.B.); giuseppe.comerci@auslromagna.it (G.C.)

**Keywords:** endometrial cancer, molecular classification, mismatch repair (MMR) status, immunotherapy

## Abstract

The therapeutic landscape of advanced and recurrent endometrial cancer (EC) has evolved substantially in recent years due to the integration of molecular classification and novel systemic therapies. This review summarizes current treatment strategies in advanced EC, focusing on immunotherapy, targeted therapies, and molecularly guided approaches. Immune checkpoint inhibitors (ICIs) have become a cornerstone of treatment, particularly in mismatch repair-deficient (dMMR)/microsatellite instability-high (MSI-H) tumors, where durable clinical benefit has been observed. Recent phase III trials demonstrated that the addition of ICIs to platinum-based chemotherapy significantly improves progression-free survival in the first-line setting, especially in dMMR disease, with more modest but clinically meaningful benefit in mismatch repair-proficient (pMMR) tumors. In the post-platinum setting, combinations such as pembrolizumab plus lenvatinib have expanded treatment options for pMMR patients, despite increased toxicity. Advances in molecular profiling, including the ProMisE classification, are increasingly guiding treatment personalization. Emerging therapies, including PARP inhibitors and antibody–drug conjugates targeting HER2 and Trop-2, are showing promising activity. Despite these advances, challenges remain regarding resistance mechanisms, optimal treatment sequencing, and predictive biomarkers beyond MMR status.

## Simple Summary

Advanced and recurrent endometrial cancer treatment has changed significantly in recent years thanks to molecular classification and the development of new targeted therapies. Immunotherapy, particularly immune checkpoint inhibitors, has shown important clinical benefits, especially in patients with mismatch repair-deficient (dMMR) or microsatellite instability-high (MSI-H) tumors. Recent clinical trials demonstrated that combining immunotherapy with standard chemotherapy improves outcomes in the first-line setting, while combinations such as pembrolizumab plus lenvatinib provide new options for patients with mismatch repair-proficient (pMMR) disease after platinum-based therapy. Molecular profiling systems, including ProMisE classification, are increasingly helping to personalize treatment strategies. In addition, emerging approaches such as PARP inhibitors and antibody–drug conjugates targeting HER2 or Trop-2 are showing promising activity. However, important challenges remain, including treatment resistance, optimal sequencing strategies, and the identification of reliable predictive biomarkers.

## 1. Introduction

Endometrial cancer (EC) represents the most common gynecological malignancy in high-income countries, with the highest incidence observed in Northern America and other regions characterized by high socioeconomic development. According to the Surveillance, Epidemiology, and End Results (SEER) Program of the National Cancer Institute, EC is the most frequent uterine neoplasm and a major contributor to cancer-related morbidity among women in Western countries. In 2025, approximately 69,120 new cases were expected, accounting for 3.4% of all newly diagnosed cancers, with an estimated 13,860 deaths attributable to this disease. The overall 5-year relative survival rate is 81.1% (2015–2021), although outcomes vary substantially according to the stage at diagnosis [1]. Endometrial cancer is associated with an overall 5-year survival of approximately 80–85%; however, mortality continues to rise in several countries, with a U.S. mortality rate of about 5–6 deaths per 100,000 women annually [2].

Most EC cases are diagnosed at an early stage, with nearly 80% of patients presenting with stage I disease, which is associated with a 5-year survival exceeding 95%. However, even among early-stage tumors, prognosis is heterogeneous and strongly influenced by histological subtype and tumor grade. High-grade endometrioid carcinomas and serous histologies exhibit less favorable outcomes, with disease-specific 5-year survival rates of approximately 86% and 74%, respectively. Notably, patients with uterine serous or clear cell carcinomas have poorer survival than those with grade 3 endometrioid tumors, even after adjustment for stage [3]. In advanced disease, prognosis markedly declines, with 5-year survival rates of approximately 68% for regional disease and 17% for distant metastatic disease [4].

From a demographic perspective, EC predominantly affects postmenopausal women, with over 90% of cases occurring after the age of 50 and a median age at diagnosis of approximately 63 years. Nonetheless, a small but clinically relevant proportion of patients—around 4%—are diagnosed before the age of 40, raising important considerations regarding fertility preservation and long-term survivorship [5].

Obesity represents the most significant modifiable risk factor for EC and is strongly associated with its increasing incidence worldwide. Excess adiposity promotes endometrial carcinogenesis through multiple mechanisms, including increased peripheral estrogen production, insulin resistance, chronic low-grade inflammation, and dysregulation of key metabolic signaling pathways [6]. Additional risk factors include prolonged exposure to endogenous or exogenous estrogens, as seen in early menarche, late menopause, or tamoxifen use. Hereditary cancer syndromes, such as Lynch syndrome and Cowden syndrome, are also associated with a markedly increased lifetime risk of EC. Furthermore, emerging evidence suggests a potential contribution of pathogenic germline BRCA1/2 variants, particularly in high-grade histologies [7].

In 1983, Bokhman described a clinicopathologic classification of EC dividing tumors into Type I and Type II [8]. This model distinguishes two pathogenetic types: Type I tumors are typically estrogen-dependent, associated with obesity and hormone receptor positivity, and generally confer a more favorable prognosis compared to Type II tumors, which are predominantly serous, more common in older non-obese women, and characterized by aggressive clinical behavior and poorer outcomes [9]. At the molecular level, Type I tumors frequently harbor alterations in Phosphatase and TENsin Homolog (PTEN), Phosphatidylinositol-4,5-bisphosphate 3-kinase catalytic subunit alpha (PIK3CA), and Kirsten Rat Sarcoma viral oncogene homolog (KRAS), and they often exhibit microsatellite instability, generally associated with a favorable prognosis. Type II tumors, including serous and clear cell carcinomas, are estrogen-independent, arise in atrophic endometrium, and are more frequently associated with TP53 mutations and chromosomal instability, leading to a more aggressive clinical course and poorer survival [10].

A major paradigm shift in the understanding of EC biology was introduced by the Cancer Genome Atlas (TCGA), which identified four widely accepted molecular subtypes: POLE ultra-mutated, mismatch repair-deficient (MMRd), p53 wild-type/copy number-low (p53 wt), and p53-mutated/copy number-high (p53abn) [9]. These molecular categories have distinct prognostic and therapeutic implications, with the POLE subtype associated with excellent outcomes and the p53abn subtype with the worst prognosis [11].

In this context, the management of advanced and recurrent EC is undergoing rapid evolution. This review aims to critically examine the evolution of treatment strategies for advanced EC, encompassing chemotherapy, immunotherapy (IT), targeted agents, radiotherapy (RT), and their integration within a molecularly driven framework. Key clinical trials will be analyzed to elucidate the mechanisms of action, clinical efficacy, and safety profiles across treatment settings. Finally, future perspectives will be discussed, with particular emphasis on treatment personalization through molecular stratification, biomarker-driven decision-making, and the integration of novel therapeutic combinations.

## 2. Molecular Classification of EC

Historically, EC has been classified according to histopathologic characteristics, integrating tumor histotype, grade, and FIGO stage. Although clinically useful for decades, the Bokhman binary system has progressively demonstrated significant limitations in prognostic discrimination and therapeutic guidance. In particular, many high-grade tumors display overlapping morphologic and clinical features that hinder accurate classification within the Type I/Type II paradigm. Conventional risk stratification therefore evolved to incorporate a combination of clinicopathologic parameters, including depth of myometrial invasion, lymphovascular space invasion (LVSI), cervical stromal involvement, and nodal status. Although these factors remain clinically relevant, they are insufficient to fully capture the biological heterogeneity of EC, thereby highlighting the intrinsic limitations of morphology-based classification systems and reinforcing the need for biologically driven stratification models [12]. This paradigm shift has progressively moved EC classification from morphology-based risk estimation toward molecularly-informed prognostic and therapeutic stratification. A major breakthrough in EC biology was provided by the TCGA Research Network, which identified four distinct molecular subgroups defined by specific mutational landscapes, copy-number alterations, and clinical outcomes. This molecular taxonomy has subsequently been translated into clinical practice through European Society of Gynaecological Oncology (ESGO), the European Society for Radiotherapy and Oncology (ESTRO), and the European Society of Pathology (ESP) guidelines—most recently updated in 2025—and now represents the cornerstone of contemporary risk stratification. Within this framework, four biologically and clinically distinct categories are recognized: POLE-ultra-mutated tumors, MMRd/microsatellite instability-high tumors (MSI-H), p53abn (copy number-high, CN-H) carcinomas, and p53 wt, also referred to as no specific molecular profile (NSMP) [9] [Table 1].

### 2.1. POLE-Ultra-Mutated Subtype

The POLE-ultra-mutated subtype is defined by pathogenic mutations within the exonuclease (proofreading) domain of the DNA polymerase epsilon gene and accounts for a relatively small (approximately 7.3%), yet clinically relevant, proportion of EC. These tumors are characterized by an ultra-high mutational burden and distinctive mutational signatures [9]. The clinical applicability of this subgroup has been consolidated through the development and validation of surrogate molecular classifiers, including the ProMisE system, now widely implemented in routine diagnostic practice [13,14]. From a clinical standpoint, POLE-mutated tumors are consistently associated with excellent oncologic outcomes, as demonstrated in integrated analyses of the PORTEC trials [15]. This favorable prognosis aligns with distinctive biological features, including dense tumor-infiltrating lymphocytes and a highly immunogenic tumor microenvironment driven by extensive neoantigen generation [13,16,17]. Notably, this favorable biology may coexist with high-grade morphologic features that, in the absence of molecular data, would otherwise classify these tumors as high risk [18]. Importantly, POLE mutations function as dominant prognostic determinants, frequently overriding adverse clinicopathologic features in therapeutic decision-making. Consistent with these data, ESGO–ESTRO–ESP guidelines recommend routine POLE testing within molecular classification algorithms [4].

### 2.2. dMMR/MSI-H

Beyond POLE-ultra-mutated tumors, the TCGA framework identifies a second hypermutated subgroup represented by MMRd/MSI-H. In this context, genomic instability arises from functional impairment of the DNA MMR system responsible for correcting replication-associated errors. The core proteins, MLH1, MSH2, MSH6, and PMS2, function as heterodimeric complexes involved in mismatch recognition and repair activation; their inactivation—secondary to somatic or germline mutations or epigenetic silencing, most commonly MLH1 promoter methylation—leads to MSI development [16,19]. This subtype accounts for approximately 25–30% of EC and is associated with a high mutational burden, intermediate between POLE-ultra-mutated and copy number-high tumors [11,20]. From a diagnostic perspective, assessment relies primarily on immunohistochemistry (IHC) demonstrating loss of nuclear protein expression, with complementary MSI-PCR or NGS testing distinguishing MSI-H from MSS tumors [21,22,23].

International recommendations support integrated morpho-molecular testing strategies given their prognostic and therapeutic implications [24,25]. Clinically, MMRd/MSI-H carcinomas display an intermediate prognostic profile between POLE-mutated and p53abn [20]. Despite prominent tumor-infiltrating lymphocytes, outcomes remain less favorable than ultra-mutated cancers. Within ESGO–ESTRO–ESP frameworks, most MMRd tumors are categorized as intermediate risk, with upstaging in the presence of substantial LVSI or deep myometrial invasion [4]. Immune checkpoint blockade efficacy in advanced disease is supported by robust clinical trial evidence [26].

### 2.3. p53abn (Copy Number-High) Subtype

Within the TCGA framework, the p53abn (copy number-high, CN-H) subtype represents a biologically aggressive category characterized by marked chromosomal instability, extensive somatic copy-number alterations, and a high prevalence of TP53 mutations [9]. This group is predominantly composed of serous carcinomas but also includes a subset of high-grade endometrioid tumors [13,27]. Aberrant p53 IHC patterns—overexpression, null staining, or cytoplasmic localization—serve as reliable surrogates of TP53 mutation. Standardized interpretation criteria have improved diagnostic reproducibility despite occasional subclonal heterogeneity [28,29]. Clinically, p53-abnormal carcinomas are associated with poor clinical outcomes, including higher recurrence rates and reduced OS. Molecular analyses from PORTEC-3 further confirmed their adverse prognostic significance [30]. In contrast to POLE-ultra-mutated and dMMR tumors, p53abn carcinomas typically display an immunologically “cold” tumor microenvironment, with limited immune infiltration [11,13]. Recognition of this subtype has major therapeutic implications, as these tumors are generally allocated to high-risk categories requiring intensified multimodal treatment strategies.

### 2.4. NSMP/Copy Number-Low Subtype

Within the TCGA molecular framework, the copy number-low subgroup—commonly referred to as noNSMP—represents the most prevalent category of EC, encompassing approximately 35–40% of cases. Unlike POLE-ultra-mutated and dMMR tumors, NSMP cancers lack defining genomic hallmarks such as proofreading mutations, microsatellite instability, or extensive copy-number alterations. Instead, they are characterized by relative genomic stability and an intermediate mutational burden [27,31].

Histopathologically, NSMP tumors are predominantly endometrioid and often low to intermediate grade. Recurrent alterations involve phosphoinositide 3-kinase (PI3K)/protein kinase B (AKT)/mechanistic target of rapamycin (mTOR) pathway genes—including PTEN, PIK3CA, and Phosphatidylinositol 3-kinase regulatory subunit alpha (PIK3R1)—as well as Catenin beta 1 (CTNNB1) mutations [32,33]. Clinically, NSMP carcinomas display intermediate outcomes but with substantial intragroup heterogeneity. Additional biomarkers—including CTNNB1 exon 3 mutations and L1CAM expression—may further refine prognostic stratification [29,31]. From a therapeutic standpoint, treatment allocation remains largely guided by clinicopathologic risk factors within ESGO–ESTRO–ESP frameworks, although ongoing translational research is actively exploring actionable vulnerabilities [11,27].

### 2.5. Prognostic and Therapeutic Implications

**Figure 1:**
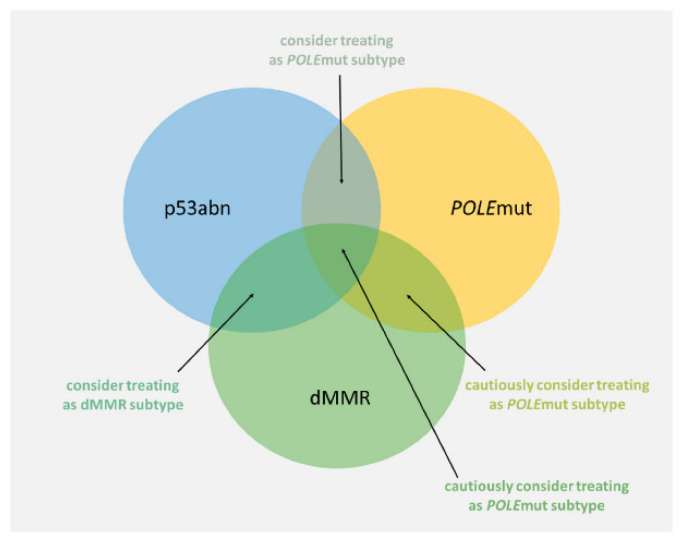
Integration of multiple molecular subtypes for the decision-making process. Adapted from [27] Accessed on 10 April 2026. Licensed under CC BY.

Collectively, the integration of POLE-ultra-mutated, dMMR, p53abn, and NSMP molecular subtypes has profoundly reshaped the prognostic landscape of EC, providing a biologically grounded framework for therapeutic stratification [34]. Beyond prognostication, this taxonomy carries direct and actionable treatment implications. Molecular profiling supports adjuvant therapy modulation, enabling de-escalation in POLE-mutated tumors with excellent outcomes, while identifying p53abn carcinomas as candidates for intensified multimodal strategies [3,15,20]. Notably, a small subset of tumors (≈3–6%) harbor multiple molecular classifiers. In such cases, hierarchical attribution is recommended, as POLE-mutated status appears to outweigh the adverse prognostic impact of concomitant p53 abnormalities, while dMMR prevails over p53 alterations. Evidence on concurrent POLE mutation and MMR deficiency remains limited and should be interpreted cautiously [27] (Figure 1). Differential sensitivity to systemic therapies has also emerged across subtypes. Hypermutated tumors—particularly dMMR cancers—demonstrate enhanced responsiveness to immune checkpoint blockade, whereas p53abn tend to display less inflamed microenvironments, supporting the development of rational combinatorial therapeutic strategies [35]. Although platinum-based chemotherapy remains the therapeutic backbone in advanced disease, molecular subtype-specific biology may significantly influence treatment responsiveness and clinical outcomes.

In routine clinical practice, molecular classification is increasingly integrated into treatment sequencing algorithms together with histologic and clinicopathologic features. In advanced or recurrent disease, dMMR/MSI-H tumors are preferentially considered for immune checkpoint inhibitor-based strategies, whereas p53abn carcinomas generally require more aggressive multimodal approaches, often including platinum-based chemotherapy combinations. POLE-mutated tumors, despite occasionally presenting with high-grade histologic features, maintain an excellent prognosis and may represent candidates for treatment de-escalation strategies. Conversely, therapeutic decision-making in NSMP tumors remains more challenging because of their biological heterogeneity and the absence of validated predictive biomarkers, thus supporting ongoing research on CTNNB1, L1CAM, homologous recombination deficiency, and PI3K/AKT/mTOR-targeted approaches.

Importantly, real-world implementation may be complicated by tumors harboring multiple molecular classifiers, intratumoral heterogeneity, or mixed histology. In these settings, hierarchical interpretation according to current ESGO–ESTRO–ESP recommendations is generally adopted, although prospective validation and standardized sequencing algorithms are still lacking.

### 2.6. Emerging Multi-Omics and Spatial Approaches

Beyond the conventional molecular classification based on the POLE-ultra-mutated, dMMR/MSI, p53-abnormal, and NSMP subtypes, emerging multi-omics approaches are progressively reshaping the biological understanding of EC and its intratumoral heterogeneity. The integration of genomics, transcriptomics, proteomics, metabolomics, spatial transcriptomics, and single-cell sequencing enables a more comprehensive characterization of tumor biology and the tumor microenvironment (TME), improving prognostic stratification and supporting the identification of potential therapeutic vulnerabilities [36]. In particular, single-cell and spatially resolved technologies can capture heterogeneity within both malignant epithelial components and immune/stromal populations, helping to characterize exhausted T cells, M2-like macrophages, immunosuppressive Tregs, and cellular networks involved in immune evasion and tumor progression [37]. Recent integrated analyses combining single-cell RNA sequencing and spatial transcriptomics have further demonstrated that the spatial architecture of the tumor microenvironment may influence the response to anti-PD-1 therapy in EC, highlighting regional immunosuppressive niches and immune cell interactions associated with therapeutic resistance [38]. In parallel, spatial TME analyses performed through imaging mass cytometry and eco-structural modeling have demonstrated that the spatial organization of immune, stromal, and endothelial cells may represent an important biological determinant of prognosis and treatment response, enabling the identification of immunosuppressive niches associated with recurrence risk and tumor aggressiveness [39]. In this context, CD90+/CD105+ endothelial cell populations have emerged as relevant components of the EC ecosystem, potentially contributing to immune infiltration dynamics, macrophage polarization, angiogenesis, and tumor heterogeneity [39]. These approaches may be particularly relevant in pMMR/MSS tumors, in which the response to immune checkpoint inhibitors remains heterogeneous and cannot be fully explained by MRR status alone. Furthermore, the integration of multi-omics data with artificial intelligence tools and advanced computational models may enable more precise prognostic classification while supporting the development of dynamic predictive biomarkers for immunotherapy response and the early identification of adaptive resistance mechanisms [36,37,38,39]. Although these technologies are not yet routinely implemented in clinical practice, they represent one of the most promising translational frameworks for future biomarker-driven precision medicine strategies in advanced EC [36].

## 3. Therapeutical Approach in Advanced Disease

### 3.1. Primary Debulking Surgery (PDS) and Interval Debulking Surgery (IDS)

While the role of surgery in early-stage EC is well established, its value in stage IV disease remains controversial, particularly in patients with peritoneal metastases who may present with unresectable disease. PDS, also referred to as cytoreductive surgery, aims to reduce the intraperitoneal tumor burden in patients with intra-abdominal disease dissemination. In this setting, neoadjuvant chemotherapy followed by IDS might represent a reasonable alternative to upfront surgery for selected patients with stage IV EC and peritoneal spread, and this strategy has been explored in several retrospective series [40,41]. In the advanced or metastatic setting (FIGO stage III–IVB), PDS followed by platinum-based adjuvant chemotherapy remains the standard of care, particularly when complete macroscopic resection can be achieved, as this is consistently associated with improved survival outcomes [4,42]. When upfront surgery is not feasible or deemed inappropriate, neoadjuvant chemotherapy may be administered, with IDS considered only in patients demonstrating a response to systemic treatment. In both adjuvant and neoadjuvant settings, carboplatin plus paclitaxel remains the preferred chemotherapy backbone, as recommended by the National Comprehensive Cancer Network (NCCN) guidelines [42] and supported by the GOG-0209 trial [43]. It must be emphasized that the absence of standardized surgical criteria for the achievement of optimal debulking in advanced EC remains an important limitation and that patients with more advanced/unresectable carcinomatosis (and, therefore, with a worse prognosis) may be referred for neoadjuvant chemotherapy and IDS rather than PDS.

A recent meta-analysis comparing patients with stage IVB EC and peritoneal spread treated with PDS plus adjuvant chemotherapy versus neoadjuvant chemotherapy followed by IDS showed a modest progression-free survival (PFS) benefit, favoring the PDS approach (18.0 vs. 12.0 months, *p* = 0.028), while no clear overall survival (OS) advantage was observed [44]. Furthermore, regardless of surgical timing, complete cytoreduction emerged as the strongest determinant of outcome, with significant improvements in both PFS (18.9 vs. 6.19 months, *p* < 0.001) and OS (40.6 vs. 21 months, *p* = 0.028). Consistently, Eto et al. reported comparable OS between patients undergoing neoadjuvant chemotherapy followed by IDS and those treated with PDS, while other retrospective studies similarly failed to demonstrate significant survival differences between strategies when optimal cytoreduction was achieved [45]. The prognostic relevance of residual disease is well established in EC with peritoneal involvement. A systematic review and meta-analysis by Albright et al. demonstrated that both submaximal and suboptimal cytoreduction were associated with significantly worse PFS and OS in patients with stage III–IV disease [46]. In this context, neoadjuvant chemotherapy should be considered in patients in whom optimal cytoreduction is unlikely, with the goal of achieving no residual disease at IDS while maintaining acceptable morbidity. Supporting this approach, Kanno et al. showed that achieving complete intra-abdominal resection—either before or after chemotherapy—was associated with prolonged survival in stage IVB EC [47]. In line with these observations, the analysis by Caiazzo et al. [48] found no statistically significant differences in OS or DFS between patients treated with neoadjuvant chemotherapy followed by IDS and those undergoing PDS followed by adjuvant chemotherapy with comparable surgical morbidity between the two strategies. Stratification by molecular subgroups did not reveal differences in oncologic outcomes, although a higher prevalence of p53-mutated tumors was observed, likely reflecting their association with biologically aggressive, advanced-stage, disease. Taken together, these results suggest that PDS should be considered the preferred treatment approach for advanced EC with intra-abdominal peritoneal spread when complete cytoreduction is achievable. However, in cases deemed unresectable at presentation, a neoadjuvant approach followed by IDS represents a valid and evidence-supported alternative. The integration of molecularly guided therapies—particularly IT in dMMR/MSI-H tumors—may improve tumor downstaging, expand surgical eligibility, and optimize cytoreductive outcomes. 

### 3.2. Chemotherapy

The historical control arm of Gynecology Oncology Group (GOG) treatment trials in women with measurable stage III–IV and recurrent EC was cytotoxic chemotherapy using doxorubicin (60 mg/mq) and cisplatin (50 mg/mq, AP) for seven cycles [49,50,51]. Subsequently, GOG0177 compared the AP regimen with TAP (doxorubicin 45 mg/mq, cisplatin 50 mg/mq, and paclitaxel 160 mg/mq) and found that TAP was associated with prolonged PFS (8 vs. 5 months) and OS (15 vs. 12 months) [50]. Since the regimen with carboplatin and paclitaxel (TC) was found to be effective in ovarian cancer, many phase II studies evaluated the same combination in EC with reported response rates of 45–78% [52,53]. Furthermore, the TAP regimen was hindered by questionable tolerability and scheduling. Therefore, the phase III GOG0209 randomized trial investigated TC regimen as a noninferior alternative to TAP, and since its noninferiority was concluded for both OS and PFS, the TC regimen became the global first standard line for advanced EC [54]. Many strategies in advanced EC have then focused on enhancing the carboplatin–paclitaxel backbone with targeted or metabolic agents. Among these, Metformin has been investigated due to its potential anticancer effects, including insulin-lowering activity, which is biologically relevant in a tumor strongly associated with obesity [55]. In parallel, the randomized phase II GOG 86P trial evaluated combinations including Bevacizumab and Temsirolimus, showing similar response rates across arms but a trend toward improved outcomes with bevacizumab-containing regimens, with a significant OS advantage observed only in the carboplatin-paclitaxel-bevacizumab arm [56]. Consistently, the smaller MITO END-2 study demonstrated that adding bevacizumab to carboplatin-paclitaxel improved response rates and PFS compared to chemotherapy alone, supporting a potential role for antiangiogenic strategies in this setting [57].

### 3.3. RT

In advanced EC, RT plays a central role in multimodal management of the disease, primarily contributing to locoregional control. According to NCCN guidelines [42], treatment includes systemic therapy with or without external beam radiotherapy (EBRT), with or without vaginal brachytherapy (VBT), based on evidence from multiple randomized trials [42] EBRT represents the backbone of radiation treatment, targeting the pelvis and, when indicated, para-aortic nodal regions, and is typically combined with systemic therapy in high-risk settings. Within this framework, VBT serves as a complementary modality aimed at reducing the risk of vaginal recurrence, which remains a common site of failure even in advanced-stage disease. Evidence from randomized trials supports the role of RT in improving locoregional outcomes. In the PORTEC-3 trial, the addition of chemotherapy administered during and after EBRT, with or without VBT, did not improve 5-year OS but significantly increased failure-free survival (FFS), particularly in patients with stage III disease, serous histology, and p53abn tumors [58]. Similarly, a combined analysis of two randomized trials demonstrated improved PFS with chemoradiation and a trend toward improved OS, especially in serous and clear cell histologies [59]. In GOG-258, comparing whole-pelvic radiotherapy (WPRT) with concurrent chemotherapy versus chemotherapy alone in patients with stage III–IVA or stage I–II serous/clear cell disease, recurrence-free survival (RFS) was similar between arms; however, chemoradiation significantly reduced vaginal and nodal recurrences [60]. In clinical practice, VBT is commonly delivered as a boost following EBRT, particularly in patients with cervical stromal involvement, close or positive vaginal margins, high-risk histological subtypes (e.g., serous or clear cell), or residual microscopic disease. However, VBT alone is not sufficient in advanced-stage disease and should not replace EBRT. Overall, while the addition of RT—especially when combined with chemotherapy—improves locoregional control, its impact on OS remains limited. Therefore, treatment strategies should be individualized, integrating EBRT and VBT according to patient- and disease-specific risk factors to optimize therapeutic outcomes while minimizing toxicity.

In oligometastatic disease, stereotactic radiosurgery (SRS) and stereotactic radiotherapy (SBRT), delivered in 1 to 5 fractions, maximize the cell-killing effect of ionizing radiation while reducing radiation-induced injuries in healthy surrounding tissues. The former is delivered only to intracranial targets, whereas the latter is delivered to extracranial ones.

### 3.4. Endocrine Therapy (ET)

In advanced EC, ET represents a biologically driven, well-tolerated option for selected patients rather than a standard of care, and it has been primarily evaluated in recurrent or metastatic disease with endometrioid histology, particularly in low-grade, indolent tumors with low burden. Its rationale is based on the hormone dependence of these tumors [61], where estrogen receptor (ER) signaling promotes proliferation, while progesterone receptor (PR) activation exerts anti-proliferative effects, with crosstalk involving the PI3K/AKT/mTOR pathway influencing sensitivity and resistance. Clinical benefit is strongly associated with ER/PR co-expression, well-differentiated histology, low proliferative index, long disease-free interval, and limited metastatic spread, especially pulmonary; conversely, p53abn tumors are typically endocrine-resistant, while hormone responsiveness is enriched in copy number-low/NSMP and some MMR-deficient tumors [62,63].

From a molecular classification perspective, endocrine sensitivity is most commonly observed in tumors belonging to the NSMP subgroup and in a subset of MMR-deficient ECs, which are more frequently associated with ER/PR expression and an indolent clinical behavior. By contrast, p53-abnormal tumors are typically associated with endocrine resistance, reflecting their aggressive biology and low hormone receptor expression. These molecular features complement established clinicopathological predictors, including low-grade endometrioid histology, low proliferative index, and limited metastatic burden.

ESGO/ESMO/ESP [4] and NCCN guidelines [42] recommend ET in selected patients with hormone receptor-positive, low-grade, indolent, or low-volume disease, particularly when chemotherapy is not appropriate. Available agents include progestins (medroxyprogesterone acetate, megestrol acetate), tamoxifen (alone or alternating with progestins), aromatase inhibitors (letrozole, anastrozole, exemestane), fulvestrant, and everolimus plus letrozole, with no regimen clearly superior. Progestins remain the most studied (response rates 15–30%) [64,65] and are particularly effective in asymptomatic, ER/PR-positive disease; tamoxifen shows ~20% activity in progesterone-resistant cases but carries thromboembolic risk in combination regimens. Aromatase inhibitors demonstrate modest activity overall but improved outcomes in receptor-positive disease (PARAGON trial: 44% clinical benefit) [66], while fulvestrant shows limited responses with occasional durable stabilization [67]. The combination of everolimus and letrozole has shown clinically meaningful activity, with phase II data reporting response rates of 22–32%, PFS of 4–6 months, and OS up to 31 months, outperforming hormonal therapy alone in some settings, particularly in chemotherapy-naïve patients. Emerging combinations with CDK4/6 inhibitors (e.g., abemaciclib or palbociclib with letrozole or fulvestrant) further improve efficacy in ER-positive disease. Despite this, ET overall provides modest benefit and no consistent survival advantage in unselected populations [68], thus making patient selection crucial. In case of progression, chemotherapy is indicated, while clinical trials or best supportive care should be considered for refractory metastatic disease.

## 4. The IT Landscape in Advanced Disease

### 4.1. Integration of Chemo-Immunotherapy in the First-Line Setting

The introduction of immune checkpoint inhibitors (ICIs) into the first-line treatment of advanced or recurrent endometrial carcinoma has substantially reshaped the therapeutic landscape. Following the demonstrated activity of anti-PD-1 (programmed Cell Death Protein 1) and PD-L1 (programmed death-ligand 1) agents in dMMR/MSI-H tumors in later treatment lines and the efficacy of pembrolizumab plus lenvatinib in pMMR disease, attention has progressively shifted toward their anticipation and integration in the first-line setting in combination with the carboplatin–paclitaxel backbone.

The biological rationale for this strategy is well established: platinum-based chemotherapy can induce immunogenic cell death, enhance tumor antigen release and presentation, and remodel the tumor microenvironment through depletion of immunosuppressive cell populations, thus creating a more permissive context for PD-1/PD-L1 blockade.

### 4.2. Phase III Randomized Trials: RUBY, NRG-GY018, AtTEnd, and DUO E

Four pivotal phase III trials evaluated the addition of ICIs to first-line carboplatin–paclitaxel, consistently demonstrating a clinically meaningful and reproducible improvement in PFS across molecular subgroups, with varying degrees of OS maturity. The RUBY trial part 1 [69,70] enrolled 494 patients with primary advanced (stage III–IV) or first recurrent (≥6 months) EC, who were randomized in a 1:1 ratio to receive carboplatin–paclitaxel in combination with either dostarlimab, an anti-PD-1 agent, or placebo, followed by maintenance therapy for up to three years. In the intention-to-treat (ITT) population, the addition of dostarlimab significantly improved outcomes, with a 24-month PFS of 36.1% compared with 18.1% in the control arm (HR 0.64; *p* < 0.001) and a 24-month overall OS of 71.3% versus 56.0% (HR 0.64; *p* = 0.0021). Updated analyses confirmed the durability and consistency of the survival benefit [69]. The magnitude of benefit was greatest in the dMMR/MSI-H subgroup (HR 0.28; *p* < 0.001), while a more modest but still statistically significant improvement was observed in pMMR tumors (HR 0.76; 95% CI 0.59–0.98). Based on these findings, the European Medicines Agency (EMA) initially expanded the indication of dostarlimab to include first-line treatment in combination with carboplatin–paclitaxel for patients with primary advanced or recurrent dMMR/MSI-H disease, and more recently extended this approval regardless of MMR status, thereby including patients with pMMR tumors. The NRG-GY018 phase III trial [71] which enrolled 810 patients and prospectively stratified analyses by MMR status, confirmed a robust and clinically relevant PFS benefit with pembrolizumab in both molecular cohorts. In the dMMR population, the risk of progression or death was reduced by 70% (HR 0.30; *p* < 0.001), with the median PFS not reached versus 7.6 months in the placebo arm. In pMMR tumors, the median PFS improved from 8.7 to 13.1 months (HR 0.54; 95% CI 0.41–0.71; *p* < 0.001). PFS benefit was maintained irrespective of PD-L1 status. EMA approved pembrolizumab in combination with carboplatin–paclitaxel for the first-line treatment of adult patients with primary advanced or recurrent EC, regardless of MMR status. In Italy, reimbursement by the Agenzia Italiana del Farmaco (AIFA) has also been granted; however, current indications are more restrictive, as pembrolizumab in combination with carboplatin–paclitaxel is reimbursed in the first-line setting specifically for patients with dMMR tumors. The AtTEnd trial [72] enrolled 551 patients and investigated the efficacy of adding atezolizumab to standard chemotherapy. After a median follow-up of 26.2 months, the atezolizumab group showed a significant benefit in the dMMR group (HR: 0.36, *p* < 0.005) with a 2-year PFS of 50.4% in the atezolizumab cohort vs. 16.0% in the placebo cohort. A statistically significant benefit was also demonstrated in the overall population (HR: 0.74), but in the non-dMMR population OS was negative. The phase III DUO-E trial [73] was designed to assess the efficacy and safety of integrating durvalumab (anti-PD-L1 agent), with or without olaparib, into standard first-line chemotherapy for patients with advanced or recurrent EC. Participants were randomized into three groups: chemotherapy alone (Arm A), chemotherapy combined with durvalumab followed by maintenance durvalumab (Arm B), and chemotherapy combined with durvalumab followed by maintenance durvalumab plus olaparib (Arm C). Both experimental strategies resulted in a significant prolongation of PFS compared with chemotherapy alone, with hazard ratios of 0.71 for Arm B and 0.55 for Arm C. Although the study was not statistically powered to directly compare the two investigational arms, exploratory analyses suggest that the addition of olaparib may further improve outcomes, particularly in the pMMR subgroup. Conversely, in dMMR tumors, the incorporation of durvalumab alone appears sufficient to confer substantial clinical benefit. Across all four phase III trials, the magnitude and durability of benefit were consistently greatest in dMMR/MSI-H tumors, confirming MMR status as the strongest predictive biomarker in the first-line setting. In pMMR disease, although a statistically significant PFS advantage was observed, the magnitude of benefit was comparatively attenuated and less consistent across studies. 

Across the four pivotal phase III trials—RUBY [69], NRG-GY018 [71], AtTEnd [72], and DUO-E [73]—several key methodological differences may help to explain the observed variability in magnitude and consistency of benefit, particularly within the pMMR/MSS subgroup.

First, substantial heterogeneity exists in trial design and immunotherapy backbone. RUBY [69], NRG-GY018 [71], and DUO-E [73] all incorporated an anti-PD-1/PD-L1 agent administered concurrently with carboplatin–paclitaxel followed by a maintenance phase with continued immune checkpoint inhibition, reflecting a “continuous exposure” strategy intended to sustain immune activation beyond chemotherapy-induced priming. By contrast, AtTEnd [72] evaluated atezolizumab without a clearly differentiated dual-maintenance strategy comparable to PD-1-based studies and used a PD-L1 inhibitor rather than PD-1 blockade, which may have contributed to differences in efficacy signals across molecular subgroups.

Second, differences in maintenance-intensification strategies are particularly relevant. DUO-E [73] uniquely evaluated a triplet strategy incorporating PARP inhibition (olaparib) in maintenance, thereby targeting DNA damage response pathways in addition to immune modulation. This design distinguishes it mechanistically from RUBY [69] and NRG-GY018 [71], which relied solely on prolonged immunotherapy, and may partly explain the greater exploratory benefit observed in biologically aggressive or genomically unstable subsets, particularly within pMMR disease.

Third, biomarker stratification and molecular classification approaches were not fully uniform across trials. NRG-GY018 [71] and DUO-E [73] prospectively stratified patients by MMR status with pre-specified subgroup analyses, ensuring robust interpretation of outcomes in dMMR versus pMMR populations. RUBY [69] also included predefined molecular subgroups but was not originally powered for biomarker-driven primary endpoints. AtTEnd, while including MMR stratification, reported less consistent benefit in the non-dMMR subgroup, raising questions about potential interactions between PD-L1 inhibition and tumor immune contexture in EC.

Fourth, patient selection and disease heterogeneity may have influenced outcomes. Although all trials enrolled patients with advanced or recurrent EC, there were differences in the proportions of primary advanced versus recurrent disease, histological subtypes (including serous and carcinosarcoma representation), and baseline prognostic risk factors. DUO-E [73], for example, included a relatively high proportion of high-grade and p53-abnormal tumors, which may have enriched genomic instability and responsiveness to combination strategies such as PARP inhibition plus immunotherapy.

Finally, differences in control arms and evolving standards of care complicate cross-trial interpretation. Notably, RUBY [69], NRG-GY018 [71], and DUO-E [73] were designed in parallel during a period when chemo-immunotherapy was emerging as a novel standard, whereas AtTEnd’s negative OS trend in the non-dMMR population may partially reflect differences in subsequent therapies and evolving post-progression management [72]. Moreover, indirect comparisons are further limited by the absence of uniform crossover strategies and differences in follow-up duration across studies.

Collectively, these methodological differences highlight that, although all four trials support the clinical activity of immune checkpoint inhibition in combination with platinum-based chemotherapy, the magnitude of benefit in pMMR/MSS disease is likely influenced not only by tumor biology but also by trial design, maintenance strategy, and biomarker framework heterogeneity. This underscores the importance of cautious cross-trial interpretation and reinforces the need for harmonized biomarker-driven trial designs in future studies. A comparative overview of the afore-mentioned trials is provided in Table 2.

### 4.3. Safety Profile of Chemo-Immunotherapy Combinations

Across the four randomized phase III trials, the addition of ICIs to platinum-based chemotherapy was associated with a modest increase in treatment-related adverse events compared to chemotherapy alone, as expected with combination regimens. The incidence of grade ≥3 adverse events was broadly comparable between treatment arms in most studies, with differences primarily driven by immune-related toxicities rather than chemotherapy-associated events. Immune-related adverse events occurred more frequently in the combination arms but were predominantly grade 1–2 and manageable with established treatment algorithms. Endocrinopathies—particularly thyroid dysfunction—represented the most commonly reported immune-mediated events. Other toxicities, such as hepatitis, colitis, and pneumonitis, were observed less frequently and were generally reversible with prompt recognition and appropriate management. Rates of permanent treatment discontinuation due to immune-related adverse events remained within an acceptable range and did not appear to compromise the overall clinical benefit. Common adverse events, including nausea, alopecia, and fatigue, were reported at similar frequencies in both treatment groups and largely reflected the cytotoxic chemotherapy backbone. No new safety signals emerged beyond the known toxicity profiles of chemotherapy and PD-1/PD-L1 blockade. Overall, the safety findings reported in RUBY, NRG-GY018, AtTEnd, and DUO-E support the feasibility of integrating immune checkpoint blockade into first-line therapy for advanced EC without compromising tolerability [70,72,73,74].

### 4.4. ICI Monotherapy in dMMR/MSI-H EC

Before the integration of ICIs into first-line chemo-immunotherapy regimens, single-agent PD-1 blockade demonstrated clinically meaningful activity in previously treated patients with dMMR/MSI-H EC. This molecular subgroup, characterized by high tumor mutational burden and enhanced neoantigen load, provides a biologically favorable context for immune activation.

The phase II KEYNOTE-158 study [35] evaluated pembrolizumab in patients with advanced MSI-H/dMMR solid tumors previously treated with standard therapy, including a dedicated EC cohort. In patients with dMMR/MSI-H EC, the objective response rate (ORR) was 48% (95% CI 37–60), with complete responses observed in a subset of patients. The median duration of response was not reached at the time of primary analysis, and the median PFS was approximately 13 months. OS data were immature. Although the trial was non-randomized and therefore did not provide hazard ratios (HRs) or comparative *p*-values, the magnitude and durability of responses were considered clinically compelling and led to tissue-agnostic regulatory approval of pembrolizumab in previously treated MSI-H/dMMR tumors. Similarly, the phase I/II GARNET trial [75,76] evaluated dostarlimab in patients with recurrent or advanced EC after prior platinum-based therapy. In the dMMR/MSI-H cohort, the ORR was 43.5%, with the median duration of response not reached at the time of reporting. As in KEYNOTE-158, the absence of a control arm precluded an estimation of HRs; however, the depth and persistence of response supported regulatory approval of dostarlimab in previously treated dMMR EC. Collectively, these studies established ICI monotherapy as a standard-of-care option in pretreated dMMR/MSI-H EC, particularly in patients not previously exposed to checkpoint blockade. Current ESMO and ESGO–ESTRO–ESP clinical practice guidelines recommend single-agent anti-PD-1 therapy for patients with advanced or recurrent dMMR/MSI-H disease progressing after platinum-based chemotherapy, emphasizing that routine assessment of MMR/MSI status is mandatory to guide therapeutic decision-making in the advanced setting [77,78]. Building on the second-line setting, and in light of the substantial benefit observed with chemo-immunotherapy —particularly in patients with dMMR disease—there is increasing interest in assessing whether single-agent IT could be a viable first-line treatment option for advanced dMMR EC. The DOMENICA trial [79] is a randomized phase III study evaluating first-line dostarlimab (500 mg every 3 weeks for cycles 1–4, followed by 1000 mg every 6 weeks for up to two years) compared to standard chemotherapy, with a planned crossover. In parallel, the KEYNOTE-C93 trial [80] is investigating a similar strategy using pembrolizumab. A key limitation of both trials is that the control arm consists of conventional carboplatin–paclitaxel chemotherapy, rather than the current standard chemo-immunotherapy combination.

### 4.5. IT Strategies in pMMR/MSS Disease

While the benefit of chemo-immunotherapy is most pronounced in dMMR/MSI-H tumors, approximately 70% of patients with advanced EC harbor pMMR/MSS disease, a subgroup characterized by lower tumor mutational burden, reduced neoantigen load, and a less inflamed tumor microenvironment, all contributing to reduced sensitivity to immune checkpoint inhibition [81]. Biologically, pMMR/MSS tumors are often characterized by low baseline immune infiltration, impaired antigen presentation, and activation of oncogenic pathways such as PI3K/AKT, p53 alterations, and Wnt/β-catenin signaling, which may promote immune exclusion and resistance to immunotherapy. In addition, an immunosuppressive microenvironment enriched in regulatory T cells, tumor-associated macrophages, and myeloid-derived suppressor cells may further limit anti-tumor immune responses.

Despite these limitations, clinically meaningful activity has been observed with chemo-immunotherapy in pMMR/MSS disease. In NRG-GY018 [74], the median PFS improved from 8.7 to 13.1 months (HR 0.54), while RUBY [70] also demonstrated a significant PFS benefit in the pMMR subgroup (HR 0.76). Similarly, DUO-E [73] showed improved outcomes with durvalumab-based combinations, whereas AtTEnd [72] failed to demonstrate a clear benefit, highlighting the biological heterogeneity within the pMMR population. Importantly, pMMR/MSS disease should not be considered a single biologic entity. Emerging subclassifications—including NSMP tumors, CTNNB1-mutated cases, and hormone receptor-positive subgroups—may further refine prognostic stratification and therapeutic selection. Hormone receptor-positive tumors may retain sensitivity to endocrine therapy, whereas CTNNB1 alterations and other genomic features may influence immune exclusion and responsiveness to immunotherapy. Likewise, subsets with increased genomic instability or DNA damage response alterations may derive greater benefit from PARP inhibitor-based strategies.

Pooled analyses suggest that the addition of immunotherapy improves PFS in pMMR/MSS disease, although the magnitude of benefit remains lower than in dMMR/MSI-H tumors and a clear OS advantage has not yet been established [82]. In DUO-E [73], the addition of olaparib to durvalumab significantly improved PFS in the pMMR/MSS subgroup, supporting the hypothesis that PARP inhibition may enhance tumor immunogenicity through activation of the cGAS–STING pathway. Exploratory analyses also suggested greater benefit in serous, high-grade, and p53abn tumors [83], potentially reflecting increased genomic instability. Similarly, RUBY Part 2 demonstrated improved PFS with the addition of maintenance niraparib to dostarlimab plus chemotherapy, including in the pMMR/MSS subgroup, although these findings remain exploratory and are not yet practice-changing given the absence of a chemo-immunotherapy control arm.

Overall, these data suggest that chemo-immunotherapy has clinically relevant activity in pMMR/MSS EC, although intrinsic biological characteristics likely necessitate combinatorial approaches to achieve durable responses [70,74,83]. Consequently, treatment selection in pMMR disease remains more nuanced than in dMMR/MSI-H tumors, and carboplatin–paclitaxel alone may still represent an appropriate first-line option in selected patients with indolent disease biology, low tumor burden, or significant comorbidities. In this evolving landscape, further biologic stratification beyond MMR status will likely become increasingly important to optimize therapeutic decision-making.

### 4.6. IT in Second-Line Setting

As mentioned above, prior to the introduction of chemo-immunotherapy in the first-line setting, single-agent PD-1 blockade firstly demonstrated clinically meaningful and durable activity in previously treated patients with dMMR/MSI-H EC. Pivotal phase II studies, including KEYNOTE-158 [35] and GARNET [75,76], showed substantial and sustained response rates in this molecular subgroup, supporting its establishment as a standard treatment option in the post-platinum setting. The debatable role of ICIs in pMMR/MSS disease provided the rationale for the development of combination strategies aimed at overcoming primary resistance to IT. In this context, therapeutic development focused on biological combinations capable of reshaping the tumor microenvironment. Lenvatinib, a multikinase inhibitor targeting Vascular endothelial growth factor receptors (VEGFR1–3), fibroblast growth factor receptors (FGFR1–4), platelet-derived growth factor receptor alpha (PDGFRα), Rearranged during transfection (RET), and proto-oncogene receptor tyrosine kinase (KIT), exerts anti-angiogenic and immunomodulatory effects that may enhance T-cell infiltration and reduce immunosuppressive signaling, thereby potentiating PD-1 blockade [84].

The phase III KEYNOTE-775 trial randomized 827 patients with advanced EC previously treated with platinum-based chemotherapy to receive pembrolizumab plus lenvatinib or physician’s choice chemotherapy. In the pMMR population, the combination significantly improved PFS, with a median PFS of 6.6 months compared to 3.8 months in the chemotherapy arm (HR 0.56; *p* < 0.001). OS was also significantly prolonged, with a median OS of 17.4–18.3 months (depending on analysis cutoff) compared to 12.0–11.4 months in the control arm (HR 0.62; *p* < 0.001) [85,86].

Notably, the survival benefit was observed irrespective of PD-L1 expression, reinforcing the limited predictive value of PD-L1 in EC [85]. Basing on these results, pembrolizumab plus lenvatinib became a standard second-line therapy for patients with pMMR/MSS advanced EC progressing after platinum-based chemotherapy [4,78]. The efficacy of pembrolizumab plus lenvatinib must be considered in the context of a substantially higher toxicity burden compared to chemotherapy alone. In KEYNOTE-775, grade ≥3 treatment-related adverse events occurred in approximately 88% of patients receiving the combination, with hypertension, diarrhea, fatigue, hypothyroidism, decreased appetite, and weight loss among the most frequent toxicities. Dose reductions of lenvatinib were commonly required, underscoring the need for careful patient selection and proactive toxicity management. Despite this toxicity profile, treatment discontinuation rates remained acceptable in view of the clinically meaningful survival benefit observed. The LEAP-001 [87] was a randomized phase III study evaluating lenvatinib plus pembrolizumab versus standard carboplatin–paclitaxel chemotherapy as first-line treatment for patients with advanced or recurrent EC. Despite strong biological rationale based on prior activity of the combination in the post-platinum setting, the trial did not meet its primary endpoints of PFS and OS in either the overall population or the pMMR subgroup. Notably, both in all-comer and pMMR populations, the combination benefit resulted in improved OS in post-adjuvant subgroups. Overall, LEAP-001 did not support replacing chemotherapy with lenvatinib–pembrolizumab in the unselected or pMMR population in the first-line setting.

### 4.7. IT in Combination with Radiotherapy

Emerging evidence supports a potential synergistic interaction between RT and immune checkpoint inhibition in EC. RT may enhance anti-tumor immunity through induction of immunogenic cell death, increased neoantigen release, improved antigen presentation, and modulation of the tumor microenvironment, thereby potentially sensitizing tumors to PD-1/PD-L1 blockade [88,89]. Preclinical and translational studies have suggested that RT may promote T-cell priming and recruitment, providing the biological rationale for combining RT with ICIs and potentially inducing systemic immune responses beyond the irradiated field (the so-called “abscopal effect”) [90].

Although prospective data in advanced EC remain limited, currently available clinical evidence suggests that the integration of RT and ICIs is generally feasible and associated with an acceptable safety profile, without major unexpected toxicities beyond those associated with each modality alone. In clinical practice, RT may be particularly relevant in selected scenarios requiring local disease control, including symptomatic bulky pelvic disease, oligometastatic or oligoprogressive disease, isolated nodal relapse, or palliation of bleeding and pain during systemic therapy. In these settings, RT may be delivered sequentially or, in selected cases, concurrently with immunotherapy, although the optimal timing, dose, and fractionation strategy remain undefined.

Interest in combined RT–immunotherapy approaches is also supported by emerging prospective clinical data. The phase III KEYNOTE-B21/ENGOT-en11/GOG-3053 trial evaluated adjuvant pembrolizumab plus chemotherapy, with radiotherapy administered according to investigator choice, in patients with newly diagnosed high-risk EC following surgery [91]. Although the addition of pembrolizumab did not significantly improve DFS in the overall population, exploratory subgroup analyses suggested a greater benefit in patients with dMMR tumors. In parallel, the ongoing phase III NRG-GY020 trial is investigating the addition of pembrolizumab to adjuvant pelvic RT in patients with high-intermediate-risk dMMR EC, with the aim of determining whether RT-induced immune priming may enhance the efficacy of PD-1 blockade in earlier-stage disease [92]. Additional early-phase and translational studies are currently exploring RT–ICI combinations in recurrent and metastatic EC, particularly in oligometastatic settings where SBRT may potentiate systemic immune responses. Overall, while RT remains primarily a locoregional treatment modality, its integration with immunotherapy represents an evolving therapeutic strategy that may further enhance systemic immune activation and disease control in selected patients with advanced EC. However, prospective studies are still needed to better define optimal patient selection, sequencing strategies, radiation parameters, and biomarkers predictive of benefit from combined RT–ICI approaches.

## 5. Predictive Biomarkers in EC

Predictive biomarkers in EC can be broadly classified into clinically actionable biomarkers, which currently inform routine therapeutic decision-making, and emerging or exploratory biomarkers, which remain under investigation and are not yet integrated into standard clinical practice. Among clinically actionable biomarkers in EC, MMR/MSI status represents one of the most robust and clinically established factors., MMR/MSI status represents one the most robust and clinically actionable factors. Clinical evidence has consistently shown that patients with dMMR tumors derive significant benefit from PD-1/PD-L1 blockade, establishing MMR status as a key determinant in therapeutic decision-making [27]. This predictive value is largely driven by the immunogenic phenotype of MMRd tumors, characterized by high mutational burden and neoantigen generation [93]. However, tumor mutational burden alone does not fully explain the heterogeneity in response. Increasing evidence suggests that qualitative features of the neoantigen repertoire, such as clonality, together with the broader immune contexture, are critical determinants of sensitivity to IT [94]. In this context, the tumor microenvironment plays a central role: inflamed (“hot”) tumors with high T-cell infiltration and interferon pathway activation are more likely to respond, whereas immunosuppressive or T-cell-excluded phenotypes may confer resistance despite dMMR [95,96].

Another clinically actionable biomarker is represented by HER2 status. HER2 overexpression and/or gene amplification are observed in approximately 25–30% of uterine serous carcinomas and in up to 10–15% of endometrial carcinosarcomas and are therefore associated with more aggressive disease features and poorer clinical outcomes. Despite their unfavorable prognostic significance, these alterations represent clinically actionable and therapeutically relevant targets. In HER2-directed therapies, the initial assessment is performed via IHC; for IHC 2+ cases, FISH (fluorescence in situ hybridization) is necessary to determine amplification. 

Among emerging or exploratory biomarkers, PD-L1 has been extensively investigated. PD-L1 expression on tumor and immune cells reflects an adaptive immune resistance mechanism and is frequently enriched in tumors with a pre-existing inflamed microenvironment. In endometrial carcinoma, PD-L1 expression is highly heterogeneous, with reported prevalence ranging from approximately 25% to 70%, depending on methodological variables and intrinsic molecular heterogeneity [97,98]. Despite its biological relevance, PD-L1 has limited utility as a standalone predictive biomarker. Clinical responses to PD-1/PD-L1 inhibitors occur irrespective of PD-L1 expression status, including in PD-L1–negative tumors, indicating suboptimal sensitivity and specificity. Moreover, its prognostic significance remains inconsistent across studies [99]. Indeed, PD-L1 assessment across the major phase III trials in advanced EC has been heterogeneous, limiting its clinical applicability as a predictive biomarker. In the RUBY and NRG-GY018 trials, PD-L1 expression was evaluated using the combined positive score (CPS), which incorporates both tumor and immune cell staining. By contrast, the DUO-E trial assessed PD-L1 expression based on tumor cell (TC) staining, typically defined by the percentage of PD-L1-positive tumor cells, without integrating immune cell components. Similarly, the AtTEnd trial used a tumor area-based scoring approach, in which PD-L1 positivity was determined by the proportion of tumor area occupied by PD-L1-expressing tumor and/or immune cells, reflecting a different methodological framework compared to CPS. This variability in assays and scoring systems, along with the lack of consistent predictive value across studies, underscores the limited role of PD-L1 as a biomarker in EC. Unlike MMR/MSI status, PD-L1 expression has not reliably identified patients most likely to benefit from immune checkpoint inhibition and is therefore not routinely used to guide treatment decisions in this setting. Based on currently available evidence, routine PD-L1 testing for therapeutic selection in endometrial cancer cannot presently be recommended. Future efforts should focus on methodological standardization and direct comparative evaluation of different PD-L1 scoring systems—including CPS, tumor cell scoring, and tumor area-based approaches—to better clarify their biological and clinical relevance.

Homologous recombination repair (HRR) deficiency represents an emerging predictive biomarker in EC. HRR deficiency, resulting in homologous recombination deficiency (HRD), is observed in a substantial proportion of ECs, particularly in high-grade and non-endometrioid histologies, and is associated with increased genomic instability [100]. Alterations in key HRR genes, including *BRCA1/2*, *ATM*, and *PALB2*, may confer increased sensitivity to platinum-based chemotherapy and PARP inhibitors, supporting their role as potential predictive biomarkers [101]. HRR alterations appear more frequently in aggressive subtypes, such as uterine serous carcinoma, and have been associated with higher tumor mutational burden and distinct genomic features [101]. Emerging data, including post hoc analyses from trials such as DUO-E, suggest that HRD-positive populations may derive enhanced benefit from PARP inhibitor-based strategies. Although the clinical role of HRR status in EC is not yet fully established, it is increasingly being explored as a therapeutic target and stratification factor in advanced disease [101].

In the era of precision medicine, p53 has emerged as both a clinically actionable prognostic and stratification biomarker within the molecular classification of EC. Beyond traditional histology, the assessment of p53 status is now mandatory for molecular risk stratification, as it identifies the p53abn subgroup—a molecularly distinct entity characterized by extreme genomic instability and the most aggressive clinical course [35]. As a prognostic biomarker, p53 mutation (detected via IHC as a reliable surrogate) outweighs traditional grading; its presence in low-grade tumors “upstages” their biological behavior, signaling a high risk of recurrence and poor survival [102]. Furthermore, p53 serves as a predictive biomarker for therapeutic response, guiding the escalation of treatment from simple observation or radiation to intensified systemic chemotherapy [77]. In the context of advanced and recurrent EC, p53 identifies patients with the most aggressive disease biology and the highest risk of treatment failure, with a dramatic decrease in PFS and OS compared to other molecular subgroups. Beyond its prognostic weight, p53 acts as a predictive biomarker for therapeutic escalation: post hoc analyses of the PORTEC-3 trial demonstrated that patients with p53abn tumors derive a significant absolute survival benefit (over 20%) from the addition of concurrent chemotherapy to radiation, a benefit not seen as clearly in other molecular groups [103]. Furthermore, in advanced serous p53-mutated cases, p53 status often co-exists with HER2 amplification, marking a specific subset of patients who benefit from targeted therapy with trastuzumab combined with standard chemotherapy [104]. Conversely, p53wt status has recently emerged as a predictive biomarker for response to maintenance therapy with selinexor, an Exportin 1 (XPO1) inhibitor [105].

Beyond the established molecular classification, the landscape of emerging biomarkers is rapidly expanding toward novel therapeutic targets for antibody–drug conjugates (ADCs). Specifically, TROP2 and folate receptor alpha (FRα) have surfaced as pivotal predictive biomarkers for advanced or recurrent disease, identifying patients eligible for highly targeted delivery of potent cytotoxic payloads [106,107]. Their overexpression—highly prevalent in aggressive p53abn subtypes—is shifting the treatment paradigm toward personalized salvage strategies for cases resistant to standard platinum-based chemotherapy [108]. Furthermore, B7-H4 and MDM2 are emerging as specialized biomarkers that further refine the molecular landscape of EC. B7-H4 is a key immune-checkpoint biomarker frequently overexpressed in p53abn serous carcinomas; it mediates immune evasion in ‘cold’ tumors, serving as a promising target for novel ADCs [109]. Conversely, MDM2 acts as a critical biomarker in p53wt cases, where its amplification functions as an alternative mechanism for p53 inactivation. This identifies high-risk patients within otherwise favorable subgroups and provides a specific molecular rationale for the use of MDM2 inhibitors to reactivate p53 function [110]. Table 3 summarizes current available data results accounting for emerging biomarkers, associated ADC, trial phase, response rates, and current clinical availability.

### Mechanisms of Therapeutic Resistance in EC

Despite the significant advances achieved with ICIs, antiangiogenic agents, PARP inhibitors, and endocrine-based strategies, both primary and acquired resistance remain major clinical challenges in advanced EC. Increasing evidence suggests that resistance mechanisms are multifactorial and closely interconnected with tumor molecular heterogeneity and dynamic evolution of the tumor microenvironment (TME). In the context of immunotherapy, resistance has been associated with impaired antigen presentation, reduced Major Histocompatibility Complex (MHC) class I expression, interferon-signaling dysregulation, T-cell exhaustion, immune exclusion phenotypes, and enrichment of immunosuppressive cellular populations, including regulatory T cells, myeloid-derived suppressor cells, and M2-polarized macrophages [111,112]. Furthermore, inflammatory remodeling of the extracellular matrix, activation of IL-6/JAK/STAT3 and NF-κB signaling pathways, and stromal-mediated immunosuppressive mechanisms may further limit cytotoxic T-cell infiltration and contribute to adaptive immune escape [112]. Emerging evidence also highlights the role of metabolic rewiring and immunometabolic escape pathways in therapeutic resistance. In particular, activation of the IDO1–kynurenine axis has been associated with the establishment of an immunosuppressive TME through tryptophan depletion, suppression of CD8+ T-cell activity, and promotion of Treg-mediated immune tolerance, representing a potential mechanism of both intrinsic and acquired resistance to immunotherapy [113]. In parallel, intratumoral heterogeneity and adaptive clonal evolution may promote the emergence of resistant cellular subpopulations during treatment exposure, particularly in pMMR/MSS tumors, where sensitivity to immune checkpoint blockade remains highly variable [37]. Similar adaptive resistance mechanisms have also been described for PARP inhibitors, endocrine therapies, and antiangiogenic agents, including restoration of homologous recombination proficiency, replication fork stabilization, compensatory activation of alternative pro-angiogenic pathways, stromal remodeling, epigenetic reprogramming, estrogen receptor signaling alterations, and PI3K/AKT/mTOR pathway activation, all of which may contribute to therapeutic escape and endocrine resistance in hormone receptor-positive disease [114,115,116,117].

**Figure 2:**
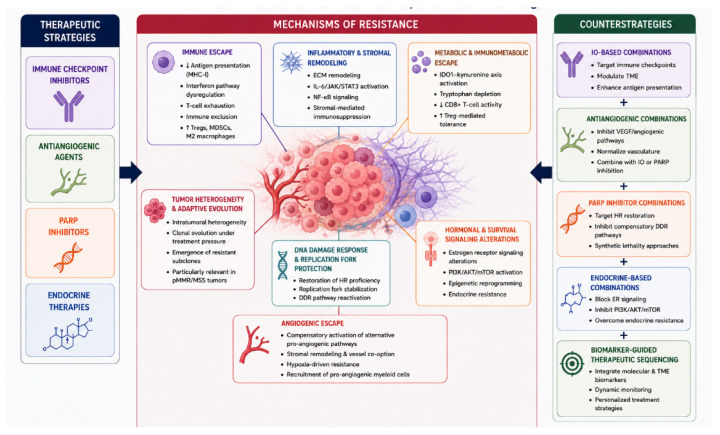
Biological mechanisms of therapeutic resistance and potential counterstrategies in advanced endometrial cancer.

Collectively, these findings support the development of a conceptual framework integrating resistance biology, tumor heterogeneity, and TME dynamics in order to guide rational combination strategies and biomarker-driven therapeutic sequencing in advanced endometrial cancer. In this context, a schematic summary of resistance pathways and corresponding therapeutic counterstrategies may provide additional translational insight and facilitate the clinical interpretation of emerging precision oncology approaches. (Figure 2).

## 6. Targeted Therapies

Targeted therapies have progressively expanded the treatment landscape of advanced EC, reflecting improved understanding of key oncogenic pathways, including angiogenesis, PI3K/AKT/mTOR signaling, and HER2 alterations. Although their activity as single agents is generally limited, their integration into combination strategies—particularly with IT—has increased their clinical relevance, as reflected in current international guidelines [4,78].

### 6.1. Angiogenesis Inhibitors

Lenvatinib is an oral multikinase inhibitor targeting VEGFR1–3, FGFR1–4, PDGFRα, RET, and KIT, exerting both anti-angiogenic and direct antitumor activity, as originally described in preclinical studies of the E7080 compound [118]. Beyond its role in vascular inhibition, lenvatinib has been shown to modulate the tumor immune environment, including reduction of tumor-associated macrophages and enhancement of T-cell infiltration, thereby supporting its use in combination strategies [119]. In EC, the clinical development of lenvatinib has been primarily driven by its combination with immune checkpoint inhibitors. As discussed above, the combination of pembrolizumab plus lenvatinib demonstrated a significant improvement in PFS and OS compared to chemotherapy in previously treated patients with advanced disease in the phase III KEYNOTE-775 trial [85], with subsequent analyses confirming the durability of OS benefit [86]. By contrast, the activity of lenvatinib as a single agent in EC appears limited, and its use is not currently supported outside combination regimens. Ongoing research is exploring its role in novel combinations and earlier lines of therapy, although results from first-line trials such as LEAP-001 have not demonstrated a clear advantage over standard chemo-immunotherapy approaches [87]. From a safety perspective, lenvatinib is associated with class-specific toxicities, including hypertension, diarrhea, fatigue, hypothyroidism, and weight loss, often requiring dose modifications and careful monitoring, as consistently reported in pivotal clinical trials [85]. Bevacizumab is a monoclonal antibody targeting vascular endothelial growth factor A (VEGF-A), thereby inhibiting tumor angiogenesis, a pathway implicated in EC progression and associated with adverse prognosis. Clinical evidence supporting bevacizumab in EC derives primarily from phase II and randomized studies evaluating its activity in recurrent or advanced disease. In a Gynaecologic Oncology Group phase II trial, bevacizumab demonstrated modest single-agent activity, with an ORR of approximately 13.5% and a median PFS of 4.2 months [120]. Subsequent studies explored bevacizumab in combination with chemotherapy. The MITO END-2 trial evaluated the addition of bevacizumab to carboplatin–paclitaxel, showing an improvement in PFS compared to chemotherapy alone, although without a clear OS benefit [57].

Overall, the clinical benefit of bevacizumab in EC appears modest, particularly when compared with more recent combination strategies involving IT. In particular, unlike lenvatinib-based regimens, bevacizumab has not demonstrated a consistent survival advantage nor a clearly defined role in current treatment algorithms. From a safety perspective, bevacizumab is associated with class-specific adverse events, including hypertension, proteinuria, thromboembolic events, and, less frequently, gastrointestinal perforation, requiring careful patient selection and monitoring. These findings suggest that VEGF inhibition as a monotherapy is insufficient to achieve durable disease control, particularly in the absence of concomitant immune modulation.

### 6.2. Targeting PI3K/AKT/mTOR

Aberrations in the PI3K/AKT/mTOR signaling pathway represent one of the most frequent molecular alterations in EC, particularly in endometrioid histology. Loss-of-function mutations in *PTEN*, activating mutations in *PIK3CA*, and alterations in *AKT* lead to constitutive pathway activation, promoting cell proliferation, survival, and metabolic reprogramming. This strong biological rationale has supported the development of targeted agents aimed at inhibiting this pathway. 

Everolimus, an oral inhibitor of mTOR, is the most extensively investigated agent targeting the PI3K/AKT/mTOR pathway in EC. Early clinical studies evaluating everolimus as a monotherapy demonstrated limited antitumor activity, with objective response rates below 10% and a median PFS of approximately 3–4 months [121]. Subsequent trials explored combination strategies, particularly with endocrine therapy, based on the known interaction between PI3K signaling and estrogen receptor pathways. A phase II study of everolimus in combination with letrozole reported an ORR of 32% and a clinical benefit rate of 40%, with a median PFS of approximately 7.4 months [122]. These findings suggest that dual targeting of hormonal signaling and the PI3K/AKT/mTOR pathway may represent a more effective strategy than pathway inhibition alone, particularly in hormone receptor-positive disease. Despite this strong biological rationale, the therapeutic targeting of the PI3K/AKT/mTOR pathway has been limited by several challenges. The pathway is characterized by complex regulatory feedback loops, whereby inhibition of mTOR can lead to compensatory activation of upstream signaling, including AKT, potentially attenuating the antitumor effect. In addition, the coexistence of multiple genomic alterations and pathway crosstalk contributes to both intrinsic and acquired resistance. From a clinical perspective, outcomes with PI3K/AKT/mTOR inhibitors in EC have remained modest overall. The absence of validated predictive biomarkers has limited appropriate patient selection, while pathway redundancy may undermine the efficacy of single-agent inhibition. Furthermore, treatment-related toxicities—including stomatitis (reported in up to 60% of patients), hyperglycemia, rash, and fatigue—can limit dose intensity and long-term tolerability. Taken together, these factors have prevented the widespread adoption of mTOR inhibitors in routine clinical practice, and current research is focused on rational combination strategies and biomarker-driven approaches.

### 6.3. HER2-Targeted Therapy

HER2 overexpression and/or gene amplification, observed in approximately 25–30% of uterine serous carcinomas and in up to 10–15% of endometrial carcinosarcomas, are associated with more aggressive disease features and poorer clinical outcomes. Despite their unfavorable prognostic significance, these alterations represent clinically actionable and therapeutically relevant target HER2-directed therapies [122,123,124]. The clinical relevance of HER2 targeting in EC was established by a randomized phase II trial (NCT01367002) evaluating the addition of trastuzumab to standard first-line carboplatin–paclitaxel backbone in patients with advanced or recurrent uterine serous carcinoma. The combination resulted in a significantly higher ORR (75% vs. 44%), as well as in a meaningful improvement in median PFS (12.9 vs. 8.0 months; HR 0.46; *p* = 0.005) and median OS (29.6 vs. 24.4 months; HR 0.58; *p* = 0.046), supporting the integration of trastuzumab into first-line treatment for HER2-positive uterine serous carcinoma [122]. More recently, the therapeutic landscape has further evolved with the development of novel HER2-targeted agents, particularly ADCs. Trastuzumab deruxtecan (T-DXd) has demonstrated promising activity across HER2-expressing solid tumors, including EC, with emerging evidence suggesting clinically meaningful responses even in heavily pretreated populations and in tumors with low or heterogeneous HER2 expression. Ongoing studies, including disease-specific cohorts within the DESTINY program, are expected to better define its role in this setting [125].

In the phase II STATICE trial, T-DXd demonstrated encouraging activity in pretreated HER2-expressing uterine carcinosarcoma, with ORRs of 54.5% in HER2-high and 70.0% in HER2-low tumors, and mPFSs of 6.2 and 6.7 months, respectively [126]. Consistent findings were reported in the phase II DESTINY-PanTumor02 trial, where T-DXd achieved an ORR of 57.5% in EC, with higher responses in HER2 3+ compared to HER2 2+ disease. Trastuzumab duocarmazine [127], another HER2-directed ADC, has also shown preliminary activity, with partial responses observed in early-phase studies, and is currently under investigation in ongoing clinical trials, both as a monotherapy and in combination strategies. Additional HER2-targeting ADCs, such as DB-1303, are being evaluated in early-phase studies, further supporting the potential role of this therapeutic class in advanced or recurrent EC.

However, several challenges remain. HER2 expression in EC is often heterogeneous, and variability in testing methodologies may hinder accurate patient selection. In addition, standardized criteria for HER2 assessment are not yet fully established, and mechanisms of resistance to HER2-targeted therapies remain not fully understood. Overall, HER2-directed therapies represent a rapidly evolving and clinically impactful strategy, particularly in the serous subtype, and exemplify the growing importance of biomarker-driven treatment approaches.

## 7. Emerging Therapeutic Strategies

Despite the significant advances achieved with chemo-immunotherapy combinations and biomarker-driven targeted therapies, outcomes in advanced endometrial carcinoma remain suboptimal, particularly in pMMR/MSS tumors and in aggressive molecular subtypes. These limitations have driven the development of novel therapeutic strategies aimed at overcoming resistance mechanisms, enhancing antitumor immunity, and expanding the spectrum of actionable targets.

### 7.1. ADCs

ADCs represent one of the most promising emerging therapeutic strategies in advanced EC, particularly in pretreated patients. These agents combine monoclonal antibody specificity with highly potent cytotoxic payloads, enabling selective drug delivery and potentially overcoming resistance to conventional therapies. For HER2-directed therapies, please refer to the dedicated Section 6.3.

Trop-2–directed ADCs, such as sacituzumab govitecan, have demonstrated clinically relevant activity in advanced EC. In the phase II TROPiCS-03 trial, conducted in heavily pretreated patients, the ORR was 22% (95% CI 11–38), with a clinical benefit rate of 32% and a median PFS of 4.8 months [106]. The median duration of response was 8.8 months, supporting durable activity in a poor-prognosis population.

The therapeutic landscape for B7-H4-expressing EC is rapidly evolving with the emergence of several promising ADCs. Among these, puxitatug samrotecan (AZD8205) and mocertatug rezetecan (GSK5733584) have demonstrated significant clinical activity in early-phase trials, with the latter showing objective response rates (ORR) as high as 67% in pretreated advanced cases [128].

Despite these encouraging signals, several important clinical limitations remain. ADCs are associated with a distinct toxicity profile that reflects both target expression in normal tissues and premature payload release in circulation. The most frequently observed adverse events include hematologic toxicity (particularly neutropenia and anemia) and gastrointestinal toxicity, which may be dose-limiting in heavily pretreated patients and can lead to treatment discontinuation. In addition, interstitial lung disease/pneumonitis has been reported with selected ADC platforms, requiring careful monitoring and early recognition.

Another key challenge is the emergence of resistance mechanisms. These include heterogeneous or downregulated target antigen expression, impaired antibody–antigen internalization, lysosomal trafficking alterations, and upregulation of drug efflux transporters, all of which may reduce intracellular delivery of the cytotoxic payload and limit durability of response.

Finally, patient selection remains a major unmet need. Although target expression (e.g., TROP2, FRα, B7-H4) is a prerequisite for ADC activity, it is not yet fully validated as a predictive biomarker in EC, and intratumoral heterogeneity may further compromise predictive accuracy. Consequently, robust biomarker-driven selection strategies and standardized companion diagnostic assays are still lacking, limiting the current clinical applicability of these agents outside clinical trials.

Table 3 summarizes current available data results accounting for emerging biomarkers, associated ADC, trial phase, response rates, and current clinical availability.

**Table 3:** Current available data results accounting for emerging biomarkers, associated ADC, trial phase, response rates, and current clinical availability.

Biomarker	Expression/Biological Relevance	ADC/Targeted Agent	Key Clinical Trial	Trial Phase	Main Efficacy Findings	Current Clinical Status
TROP2 [106]	Frequently overexpressed in aggressive EC, particularly p53abn tumors; associated with tumor proliferation and metastatic potential	Sacituzumab govitecan	TROPiCS-03	Phase II	ORR 22%; median PFS 4.8 months; median DoR 8.8 months in heavily pretreated EC	Investigational in EC; not currently approved
FRα (Folate receptor-α) [107]	Overexpressed in a subset of serous and high-grade EC; associated with aggressive biology and platinum resistance	Mirvetuximab soravtansine	Early basket/expansion studies	Phase I/II	Preliminary antitumor activity in FRα-positive EC; data still limited	Investigational; biomarker testing not standardized in EC
B7-H4 [129]	Immune checkpoint protein highly expressed in serous and p53abn EC; associated with immune evasion and “cold” tumor microenvironment	Mocertatug rezetecan (GSK5733584)	BEHOLD-1	Phase I/II	ORR up to 67% in heavily pretreated B7-H4-positive EC	Investigational
HER2 [126]	Amplified/overexpressed mainly in uterine serous carcinoma and carcinosarcoma	Trastuzumab deruxtecan (T-DXd)	DESTINY-PanTumor02	Phase II	Clinically meaningful responses in HER2-positive gynecologic tumors including EC	Emerging therapeutic option; not yet standard in EC

ADC, antibody–drug conjugate; DoR, duration of response; EC, endometrial cancer; FRα, folate receptor-α; HER2, human epidermal growth factor receptor 2; ORR, objective response rate; PFS, progression-free survival; T-DXd, trastuzumab deruxtecan; TROP2, trophoblast cell-surface antigen 2.

### 7.2. XPO1 Inhibitors

Targeting nuclear export represents a novel therapeutic strategy in advanced EC. XPO1 is frequently overexpressed in endometrial carcinoma and mediates the nuclear export of multiple tumor suppressor proteins, including p53, p73, and BRCA1; its inhibition results in nuclear retention of these proteins and activation of pro-apoptotic pathways [129]. Selinexor is an oral selective inhibitor of XPO1 that has demonstrated antitumor activity in early-phase studies, with evidence of disease control in heavily pretreated EC patients [130].

The most relevant clinical evidence derives from the randomized phase III SIENDO trial (ENGOT-EN5/GOG-3055), which evaluated selinexor as a maintenance therapy following first-line chemotherapy. In the overall population, the improvement in PFS did not reach statistical significance (median PFS 5.7 vs. 3.8 months; HR 0.76; *p* = 0.126) [114]. However, a pre-specified subgroup analysis demonstrated a substantial benefit in TP53 wild-type tumors, with a median PFS of 28.4 vs. 5.2 months (HR 0.44; *p* = 0.0005), which was further enhanced in the pMMR subgroup (39.5 vs. 4.9 months; HR 0.36; *p* = 0.0011) [131]. Overall, although selinexor did not demonstrate a statistically significant benefit in the unselected population, these data suggest a potential role in molecularly selected subgroups. Ongoing phase III trials (XPORT-EC-042) will further clarify the clinical positioning of XPO1 inhibition in EC [132].

### 7.3. Novel Endocrine-Based Strategies

Novel endocrine-based strategies in oncology are increasingly evolving toward more sophisticated approaches aimed at overcoming both primary and acquired resistance to hormone therapies. It is now well established that endocrine resistance is driven by a complex network of molecular alterations, including activation of alternative signaling pathways—particularly PI3K/AKT/mTOR and HER2—dysregulation of cell cycle control, and mutations in the estrogen receptor gene (ESR1), which promote ligand-independent receptor activation and sustained tumor proliferation [133,134,135]. In this context, the development of next-generation selective estrogen receptor degraders (SERDs), especially oral agents such as elacestrant and giredestrant, represents a major advancement, as these compounds induce degradation of both wild-type and mutant estrogen receptors and retain activity in ESR1-mutant tumors. In parallel, the integration of ET with targeted agents has substantially reshaped the therapeutic landscape. CDK4/6 inhibitors act by blocking cell cycle progression at the G1–S transition, while inhibitors of the PI3K/AKT/mTOR pathway suppress compensatory survival signaling, collectively leading to significant improvements in PFS [136]. Additional emerging strategies include the development of novel agents such as PROTACs and next-generation selective estrogen receptor modulators (SERMs), as well as the exploration of combinations with IT, supported by growing evidence of crosstalk between hormonal signaling and the tumor microenvironment. Finally, the implementation of precision medicine approaches—driven by biomarker-based patient selection and advanced technologies such as single-cell sequencing and tumor organoid models—is expected to further refine endocrine-based strategies and enable increasingly personalized and effective therapeutic interventions [137].

## 8. Treatment Algorithm Proposal

### 8.1. De-Novo Advanced EC (Figure 3)

In patients with stage III–IV EC, including carcinosarcoma, surgical cytoreduction should be considered when complete macroscopic resection is achievable with acceptable morbidity and quality of life, following comprehensive pre-operative staging and multidisciplinary discussion. Systematic lymphadenectomy is not recommended; only suspicious lymph nodes should be removed as part of the cytoreductive surgery. For patients with unresectable stage III or IV due to local extent of disease, multidisciplinary team discussions should consider the molecular subtype of the tumor in decision-making about definitive RT (with EBRT and image-guided brachytherapy) or primary systemic treatment. 

The combination of the carboplatin–paclitaxel backbone with anti-PD-1 dostarlimab in patients with dMMR status is recommended in stage IIIA–IIIC1 only in the presence of RECIST 1.1 measurable/evaluable disease; stage IIIC1 carcinosarcoma with clear cell, serous, or mixed histology regardless of measurability; and stage IIIC2–IV histologies regardless of measurability. RT can be integrated within a multimodal treatment strategy alongside chemo-immunotherapy in advanced EC. Its use is most commonly recommended as sequential or consolidative, following systemic treatment, particularly for locoregional residual disease, pelvic control, or oligometastatic sites. Concomitant administration with chemo-immunotherapy may be considered in selected clinical situations, such as symptomatic bulky pelvic disease, although evidence remains limited and this approach is not yet standardized. Overall, RT represents an important complementary modality within a multidisciplinary treatment pathway rather than a substitute for systemic chemo-immunotherapy. For pMMR disease, treatment selection is still an unmet need. Since chemo-immunotherapy with dostarlimab plus carboplatin–paclitaxel is currently an approved and guideline-supported option regardless of MMR status, this combination is suitable in this setting, although the magnitude of benefit is more modest compared to dMMR disease. Within this heterogeneous group, additional molecular and histopathological factors should be considered. In patients with high-risk features, including p53abn tumors or serous histology, treatment intensification strategies may be considered; however, combinations incorporating PARP inhibitors (e.g., durvalumab plus olaparib as investigated in DUO-E) remain investigational and are not yet standard of care.

**Figure 3:**
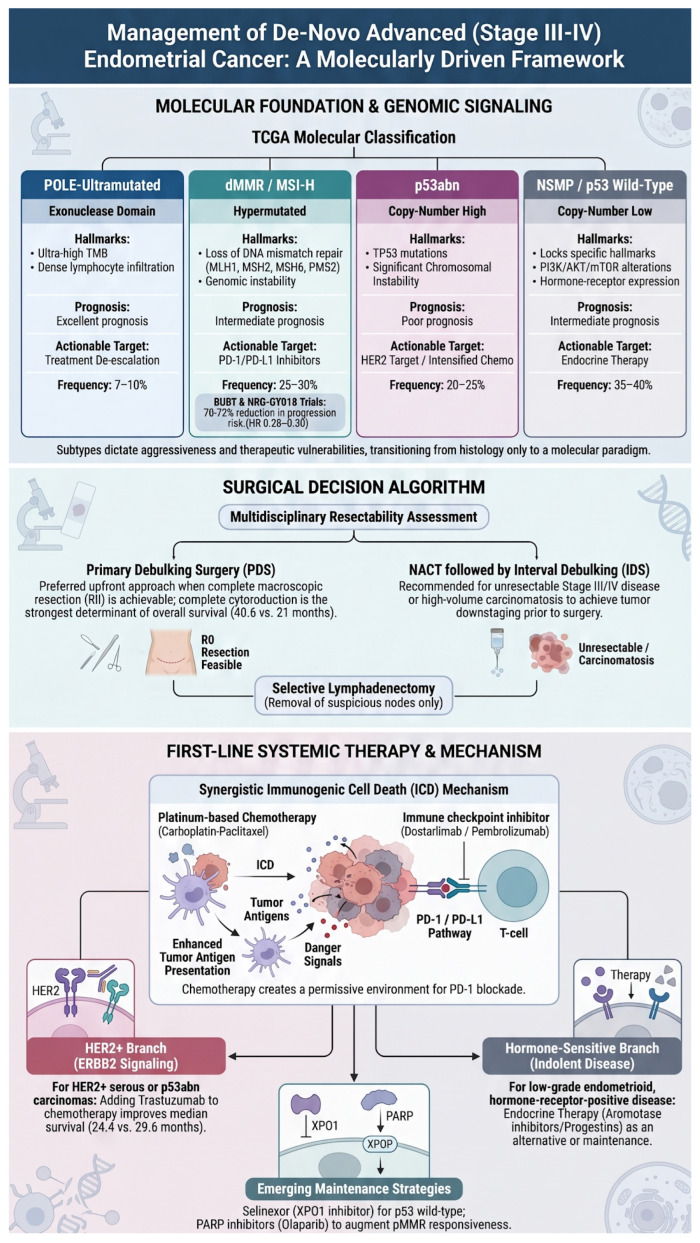
Treatment algorithm for de novo advanced EC, based on molecular classification and heterogeneous signaling pathways.

HER2 testing is recommended in serous and/or p53abn carcinomas, as the addition of trastuzumab to chemotherapy may improve outcomes in HER2-positive disease, regardless of MMR status, with this combination being approved for first-line treatment only. In Italy, trastuzumab can be prescribed and reimbursed under specific criteria, including eligibility through AIFA Note 648. Hormone receptor status should be assessed, particularly in low-grade endometrioid tumors, as ET may represent a treatment option in selected patients with indolent disease. In this setting, hormonal therapy can be used both as a monotherapy and as a maintenance strategy after response to systemic treatment. As a single-agent approach, it is mainly reserved for patients with slowly progressive, low-volume, hormone receptor-positive disease who are unfit for or wish to delay chemotherapy, with the aim of achieving disease control while minimizing toxicity. Emerging strategies, such as selinexor in p53wt tumors, are currently under clinical investigation and should be considered experimental. Overall, treatment decisions should integrate molecular classification (MMR, p53, POLE), histology, and patient-related factors, with enrolment in clinical trials strongly encouraged whenever possible, particularly for patients with pMMR disease, where unmet needs remain significant.

### 8.2. Recurrent Advanced EC (Figure 4)

In patients who relapse more than 6–12 months after prior (neo)adjuvant platinum-based chemotherapy, re-treatment with carboplatin–paclitaxel may be considered. In this context, the addition of IT can be reasonably accounted for, particularly in IT-naïve patients and regardless of MMR status, as such populations were included in first-line phase III trials. However, this approach is not formally established as a standard “first-line-like relapse” strategy and should be individualized according to symptoms, disease burden, prior treatment tolerance, and disease kinetics.

In dMMR/MSI-H tumors, single-agent PD-1 blockade (e.g., dostarlimab or pembrolizumab) represents the preferred option if not previously administered, given the high response rates and durability of benefit observed in this setting. In pMMR/MSS disease, the combination of pembrolizumab plus lenvatinib remains the standard of care after prior platinum-based chemotherapy, based on significant improvements in PFS and OS compared to chemotherapy. This regimen should be considered the preferred option in eligible patients, despite its higher toxicity profile. Alternative options include single-agent chemotherapy, particularly in patients unfit for combination therapy, although clinical benefit is generally modest. ET may be considered in selected patients with low-grade, hormone receptor-positive, indolent disease; however, it may also be used in selected frail or chemotherapy-ineligible patients with higher disease burden, provided hormone receptor expression is present.

The optimal management of patients progressing after first-line chemo-immunotherapy remains undefined, as most currently available second-line evidence derives from studies conducted in ICI-naïve populations. At present, there is no established standard regarding ICI rechallenge, continuation beyond progression, or switching to an alternative PD-1/PD-L1 inhibitor, and prospective evidence supporting these strategies is lacking. In selected pMMR/MSS patients previously exposed to ICIs, pembrolizumab plus lenvatinib may still represent a reasonable therapeutic option, although data specifically addressing efficacy after prior chemo-immunotherapy are limited. Similarly, PARP inhibitor-based combinations remain investigational in this setting and may be more relevant in biomarker-selected populations, including HRD-positive or p53abn tumors. Overall, enrolment in clinical trials should be strongly encouraged whenever feasible, given the substantial evidence gaps that continue to characterize post-chemo-immunotherapy treatment sequencing in advanced EC.

**Figure 4:**
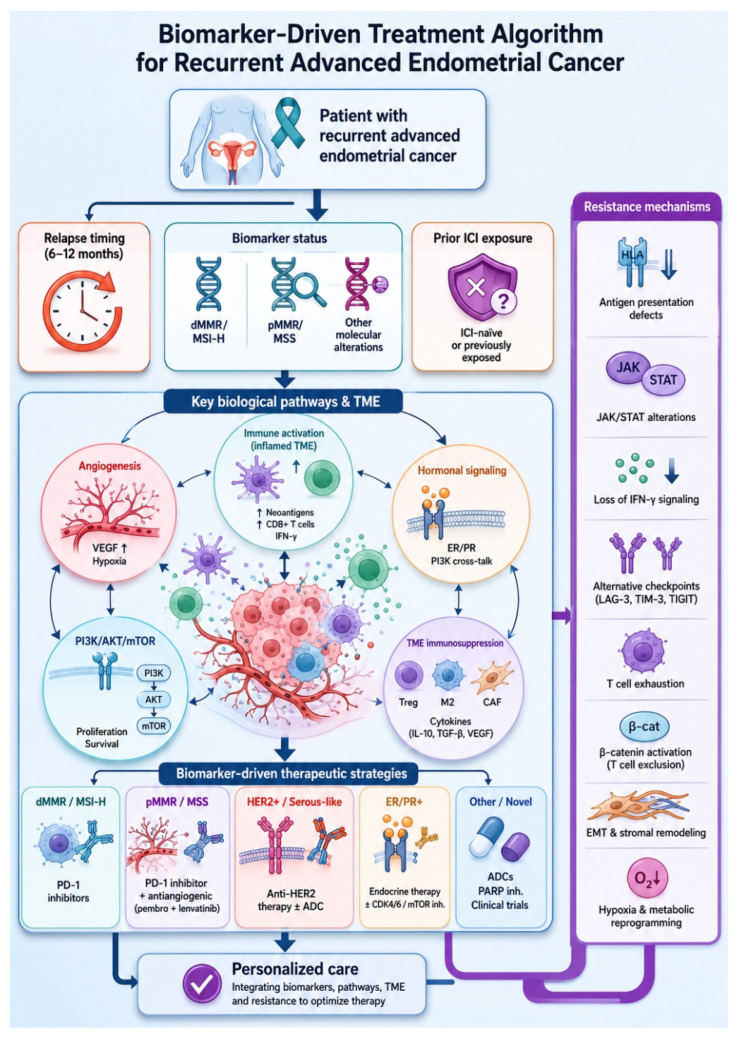
Treatment algorithm for recurrent or advanced EC.

## 9. Conclusions

In the past, therapeutic options for advanced or recurrent EC were limited; however, the advent of targeted therapies and IT has significantly reshaped the treatment landscape. Molecular classification has become central to treatment selection, enabling more tailored approaches, although tumor heterogeneity and resistance mechanisms remain major challenges. Recent phase III trials have demonstrated a clear survival benefit with the addition of IT to first-line chemotherapy, particularly in MMRd tumors, with a more modest but still meaningful effect in the MMRp population. The integration of PARP inhibitors into combination strategies has further improved outcomes, although their precise role in MMRp disease requires clarification. Ongoing efforts are focused on refining patient selection, overcoming primary and acquired resistance, and defining optimal treatment duration. The ProMisE framework provides a basis for tailoring therapy, but further molecular stratification is needed, especially for the heterogeneous MMRp subgroup. Finally, emerging therapies, such as antibody–drug conjugates, are expanding the therapeutic armamentarium and may offer additional opportunities for biomarker-driven treatment in advanced EC.

Despite the major therapeutic advances achieved with chemo-immunotherapy, several clinically relevant controversies and limitations remain unresolved in advanced EC. One of the most important open questions concerns the optimal sequencing of systemic therapies and immunotherapy across treatment lines. With the increasing adoption of chemo-immunotherapy in the first-line setting, the role of subsequent immune checkpoint inhibition after progression has become uncertain, particularly in patients previously exposed to anti-PD-1/PD-L1 agents. Currently, prospective data guiding post-chemo-immunotherapy treatment strategies are lacking, and the efficacy of ICI rechallenge or continuation beyond progression remains undefined. Similarly, the optimal management of patients progressing after first-line chemo-immunotherapy represents a major unmet clinical need. In pMMR/MSS disease, pembrolizumab plus lenvatinib remains a potential option in selected cases; however, evidence supporting its activity after prior exposure to ICIs is still limited. In dMMR/MSI-H tumors, resistance following initial immunotherapy response is increasingly recognized, but the underlying biological mechanisms and optimal salvage approaches remain poorly characterized.

More broadly, resistance mechanisms to immunotherapy and targeted agents are still not fully understood, and the heterogeneity of pMMR tumors further complicates therapeutic decision-making. Although MMR/MSI status currently represents the most clinically relevant predictive biomarker, additional biomarkers are needed to better identify patients most likely to benefit from specific therapeutic strategies. Beyond MMR status, refinement of biomarker-driven patient selection—including HRD, p53 status, TROP2, FRα, and tumor immune microenvironment features—will likely play an increasingly important role in future treatment algorithms. Furthermore, the limited availability of mature OS data from several randomized studies and the toxicity profile of some combination regimens continue to complicate treatment selection in routine clinical practice.

Another evolving area concerns the future role of personalized combination strategies. Increasing evidence suggests that molecularly tailored approaches may be required to overcome primary and acquired resistance, particularly in pMMR disease. Combinations involving PARP inhibitors, anti-angiogenic agents, endocrine therapy, CDK4/6 inhibitors, and antibody–drug conjugates are currently under active investigation, with the aim of improving tumor immunogenicity and expanding the proportion of patients deriving durable benefit from systemic therapy.

Future research should therefore focus on biomarker-driven therapeutic approaches, optimization of treatment sequencing and combination strategies, and the development of novel agents, including antibody–drug conjugates and next-generation immunotherapeutic combinations. Ongoing translational studies will likely improve the understanding of tumor biology and resistance mechanisms, ultimately contributing to a more precise and individualized treatment approach for patients with advanced EC. Overall, continued integration of molecular profiling with innovative therapeutic strategies will be essential to further improve outcomes for patients with advanced EC.

## Figures and Tables

**Table 1:** Molecular classification of endometrial carcinoma: biological, prognostic, and therapeutic implications.

Subtype	Key Features	Frequency	Prognosis	Therapeutic Implication
POLE-ultra-mutated	POLE mutations; ultra-high TMB	7–10%	Excellent	De-escalation
dMMR/MSI-H	MMR loss; MSI; hypermutated	25–30%	Intermediate	Immunotherapy
p53abn	TP53 mutations; CN-high	20–25%	Poor	Intensified therapy
NSMP	No specific profile; PI3K/CTNNB1	35–40%	Intermediate	Risk-based/research

dMMR, mismatch repair-deficient; MSI-H, microsatellite instability-high; p53abn, p53 abnormal; NSMP, no specific molecular profile.

**Table 2:** Key phase III trials of first-line chemo-immunotherapy in advanced EC.

Trial	Regimen	Population	PFS (HR)	OS (HR)	Grade ≥ 3 AEs	Discontinuation Due to AEs	QoL Findings	Key Findings
RUBY (ENGOT-EN6/GOG-3031) [69]	Carboplatin–paclitaxel + dostarlimab → maintenance dostarlimab	Advanced/recurrent EC (all-comers)	0.64 overall; 0.28 (dMMR); 0.76 (pMMR)	0.64	~63%	~17%	Generally maintained over time	Significant PFS and OS benefit; strongest effect in dMMR
NRG-GY018 [71]	Carboplatin–paclitaxel + pembrolizumab → maintenance pembrolizumab	Stratified by MMR status	0.30 (dMMR); 0.54 (pMMR)	Immature	~63–71%	~13%	Limited mature QoL data	Robust PFS benefit across both subgroups
AtTEnd (ENGOT-en7) [72]	Carboplatin–paclitaxel + atezolizumab	Advanced/recurrent EC	0.74	0.82 (NS)	~79%	~15%	Limited QoL data available	Modest PFS benefit; OS not statistically significant
DUO-E (GOG-3041/ENGOT-EN10) [73]	Carboplatin–paclitaxel + durvalumab ± olaparib	Advanced/recurrent EC	0.71 (durvalumab); 0.55 (durvalumab + olaparib)	0.77 (NS)	~73–82%	~20% (triplet arm)	Generally maintained despite increased toxicity	PFS benefit; exploratory advantage with PARP inhibition

AE, adverse event; dMMR, mismatch repair-deficient; EC, endometrial cancer; HR, hazard ratio; MMR, mismatch repair; NS, not significant; OS, overall survival; PARP, poly(ADP-ribose) polymerase; PFS, progression-free survival; pMMR, mismatch repair-proficient; QoL, quality of life.

## Data Availability

No new data were created or analyzed in this study. Data sharing is not applicable to this article.

## Abbreviations

ADCs	Antibody–Drug Conjugates
AIFA	Agenzia Italiana del Farmaco
AKT	Protein Kinase B
AP	Doxorubicin + Cisplatin
ATM	Ataxia Telangiectasia Mutated
BRCA1/2	Breast Cancer Gene 1/2
CDK4/6	Cyclin-Dependent Kinases 4 and 6
CI	Confidence Interval
CN-H	Copy-Number High
CPS	Combined Positive Score
CTNNB1	Catenin Beta 1
DFS	Disease-Free Survival
DIMEC	Department of Medical Sciences
dMMR	Deficient Mismatch Repair
DNA	Deoxyribonucleic Acid
DOACs	Direct Oral Anticoagulants
EC	Endometrial Cancer
EBRT	External Beam Radiotherapy
EMA	European Medicines Agency
ENGOT	European Network of Gynaecological Oncological Trial Groups
ER	Estrogen Receptor
ESGO	European Society of Gynaecological Oncology
ESMO	European Society for Medical Oncology
ESP	European Society of Pathology
ESR1	Estrogen Receptor 1
ESTRO	European Society for Radiotherapy and Oncology
ET	Endocrine Therapy
FFS	Failure-Free Survival
FGFR	Fibroblast Growth Factor Receptor
FIGO	International Federation of Gynecology and Obstetrics
FISH	Fluorescence In Situ Hybridization
GOG	Gynecologic Oncology Group
HER2	Human Epidermal Growth Factor Receptor 2
HPV	Human Papillomavirus
HR	Hazard Ratio
HRD	Homologous Recombination Deficiency
HRR	Homologous Recombination Repair
ICI	Immune Checkpoint Inhibitor
ICIs	Immune Checkpoint Inhibitors
IDS	Interval Debulking Surgery
IHC	Immunohistochemistry
IT	Immunotherapy
ITT	Intention-To-Treat
KIT	Proto-Oncogene Receptor Tyrosine Kinase
KRAS	Kirsten Rat Sarcoma Viral Oncogene Homolog
LVSI	Lymphovascular Space Invasion
MDM2	Mouse Double Minute 2 Homolog
MLH1	MutL Homolog 1
MMR	Mismatch Repair
MMRd	Mismatch Repair-Deficient
MSH2	MutS Homolog 2
MSH6	MutS Homolog 6
MSI	Microsatellite Instability
MSI-H	Microsatellite Instability-High
MSI-PCR	Microsatellite Instability-Polymerase Chain Reaction
MSS	Microsatellite Stable
mTOR	Mechanistic Target of Rapamycin
NCCN	National Comprehensive Cancer Network
NGS	Next-Generation Sequencing
NS	Not Significant
NSMP	No Specific Molecular Profile
ORR	Objective/Overall Response Rate
OS	Overall Survival
PALB2	Partner and Localizer of BRCA2
PARP	Poly-ADP-Ribose Polymerase
PD-1	Programmed Cell Death Protein 1
PD-L1	Programmed Death-Ligand 1
PDS	Primary Debulking Surgery
PFS	Progression-Free Survival
PI3K	Phosphoinositide 3-Kinase
PIK3CA	Phosphatidylinositol-4,5-Bisphosphate 3-Kinase Catalytic Subunit Alpha
PIK3R1	Phosphatidylinositol 3-Kinase Regulatory Subunit Alpha
pMMR	Proficient Mismatch Repair
PMS2	PMS1 Homolog 2
POLE	DNA Polymerase Epsilon
PR	Progesterone Receptor
ProMisE	Proactive Molecular Risk Classifier for Endometrial Cancer
PTEN	Phosphatase and TENsin Homolog
RECIST	Response Evaluation Criteria in Solid Tumors
RET	Rearranged During Transfection
RFS	Recurrence-Free Survival
RT	Radiotherapy
SBRT	Stereotactic Body Radiotherapy
SEER	Surveillance, Epidemiology, and End Results
SERDs	Selective Estrogen Receptor Degraders
SERMs	Selective Estrogen Receptor Modulators
SRS	Stereotactic Radiosurgery
TAP	Paclitaxel + Doxorubicin + Cisplatin
TC	Carboplatin + Paclitaxel
TCGA	The Cancer Genome Atlas
T-DXd	Trastuzumab Deruxtecan
TMB	Tumor Mutational Burden
TP53	Tumor Protein p53
Trop-2/TROP2	Trophoblast Cell Surface Antigen 2
VEGF	Vascular Endothelial Growth Factor
VEGF-A	Vascular Endothelial Growth Factor A
VEGFR	Vascular Endothelial Growth Factor Receptor
VBT	Vaginal Brachytherapy
WPRT	Whole-Pelvic Radiotherapy
XPO1	Exportin 1
